# Research and Application of Underground WLAN Adaptive Radio Fingerprint Database

**DOI:** 10.3390/s20041182

**Published:** 2020-02-21

**Authors:** Jiansheng Qian, Mingzhi Song

**Affiliations:** School of Information and Control Engineering, China University of Mining and Technology, Xuzhou 221000, China; qianjsh@cumt.edu.cn

**Keywords:** fingerprint positioning, WiFi, adaptive radio fingerprints database, ULTF, QPSO

## Abstract

Fingerprint positioning based on WiFi in coal mines has received much attention because of the widespread application of WiFi. Fingerprinting techniques have developed rapidly due to the efforts of many researchers. However, the off-line construction of the radio fingerprint database is a tedious and time-consuming process. When the underground environments change, it may be necessary to update the signal received signal strength indication (RSSI) of all reference points, which will affect the normal working of a personnel positioning system. To solve this problem, an adaptive construction and update method based on a quantum-behaved particle swarm optimization–user-location trajectory feedback (QPSO–ULTF) for a radio fingerprint database is proposed. The principle of ULTF is that the mobile terminal records and uploads the related dataset in the process of user’s walking, and it forms the user-location track with RSSI through the analysis and processing of the positioning system server. QPSO algorithm is used for the optimal radio fingerprint match between the RSSI of the access point (AP) contained in the dataset of user-location track and the calibration samples to achieve the adaptive generation and update of the radio fingerprint samples. The experimental results show that the radio fingerprint database generated by the QPSO–ULTF is similar to the traditional radio fingerprint database in the statistical distribution characteristics of the signal received signal strength (RSS) at each reference point. Therefore, the adaptive radio fingerprint database can replace the traditional radio fingerprint database. The comparable results of well-known traditional positioning methods demonstrate that the radio fingerprint database generated or updated by the QPSO–ULTF has a good positioning effect, which can ensure the normal operation of a personnel positioning system.

## 1. Introduction

The positioning of coal mine personnel is one of the important research topics in coal mine safety management [1,2]. The recording and monitoring of miners’ locations ensure the management efficiency of coal mine production and the effectiveness of an underground accident rescue. In recent years, the method of underground location fingerprint positioning based on WiFi technology has been widely studied because of its low cost, wide coverage, high transmission rate, expandability, better confidentiality, maintainability, accessibility to many users, and strong anti-interference ability [3,4,5].

At present, the research on the location fingerprint positioning of a Wireless Local Area Network (WLAN) mainly focuses on the noise reduction of radio fingerprint samples [6], the optimal selection of the wireless access point [7], and an online radio fingerprint-matching algorithm [8,9]. Beyond that, there has been a lot of research on the construction and maintenance of a radio fingerprint database in applications. It takes a good deal of time to build a radio fingerprint database manually. When the location area environment changes, which results in a change in RSS distribution, the database needs to re-collect the radio fingerprint samples of a large number of reference points. This process will seriously affect the normal use of the underground personnel positioning system [10]. To solve this problem, a new Adaptive Indoor Positioning System model (called DIPS) has been proposed in [11]. DIPS is based on a dynamic radio map generator, RSS certainty technique, and peoples’ presence effect integration for dynamic and multi-floor environments. DIPS is also able to overcome the mobile device heterogeneity problem and considers the presence of people as a WLAN signal’s attenuation factor. Wu, Yang, and Liu designed a WLAN location fingerprint system named LiFS (Locating in Fingerprint Space) [12]. The feature of LiFS is that the radio map generator does not need to allocate a lot of time to create radio maps in the off-line phase, but only needs a small number of users’ walking data recorded by a mobile device. In LiFS, the calibration of fingerprints is crowdsourced and automatic. Kim proposed an autonomous radio fingerprinting method based on cooperative feedback of mobile phones [13]. Mobile users track their position with mobile phones and measure RSS from the surrounding access points. Anonymous mobile users automatically collect data in daily life without purposefully surveying an entire positioning area. DIPS, LiFS, and the method in [13] effectively make up the drawbacks of creating radio maps. However, they are mainly used in indoor environments such as office buildings; those methods are not suitable for underground environments.

**Figure 1:**
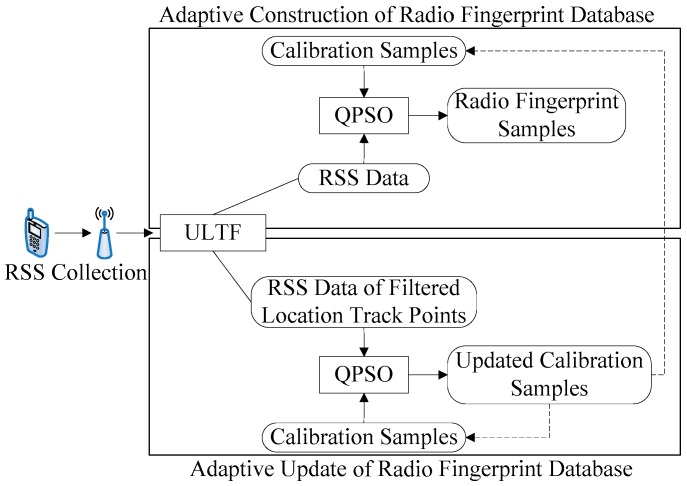
Concept map of adaptive construction and update method based on the quantum-behaved particle swarm optimization–user-location trajectory (QPSO–ULTF) algorithm.

In view of the tedious construction and maintenance of the underground WLAN radio fingerprint database, this paper proposes an adaptive construction and update method of the radio fingerprint database based on the quantum-behaved particle swarm optimization–user-location trajectory feedback (QPSO–ULTF) algorithm. The concept map is shown in Figure 1. As the miners walk through an underground tunnel, the ULTF algorithm records their relevant positions and RSS data with the technical advantages of a mobile terminal. In the process of creating a radio fingerprint database, each user-location point has its corresponding candidate reference points. The calibration samples belonging to these candidate reference points are used as a scale, and the QPSO algorithm is used to adaptively adjust the RSS data feedback by the user-location track. In the process of updating the radio fingerprint database, the RSS data of filtered user-location track points is used as a scale, and the QPSO algorithm is used to update the calibration samples of corresponding reference points. The secondary construction of the radio fingerprint samples is then used to complete the update of the radio fingerprint database. In general, the radio fingerprint database can be updated in a relatively low-frequency period of time when the miners enter or leave the location area, so as to minimize the impact of the update process on the normal operation of the location fingerprint positioning system. The experiments and analysis show that the QPSO–ULTF can effectively replace the traditional manual acquisition process in the construction and update of the radio fingerprint database. It can also adaptively complete the maintenance of the radio fingerprint database without affecting the normal operation of the positioning system. Furthermore, it reduces resource consumption and makes the system more robust.

## 2. QPSO Algorithm

QPSO [14] is a particle swarm optimization (PSO) algorithm based on the quantum behavior of particles, proposed by Sun et al. It is an important improvement of the PSO algorithm in terms of its global search ability and operational efficiency. The PSO algorithm is a cluster optimization algorithm that uses the histories of many potential optimal solutions to choose and share in the iteration. During this process, the particles continuously complete the evolution and approaches to the global optimal solution. PSO has a high complexity in the optimization problem with large dimensions. Each particle will carry out a large number of local optimal solutions in the high-dimension space, which has a significant impact on the overall performance of PSO. QPSO uses the quantum state of particles to replace the velocity of particles. It is useful to update the position of particles because this reduces the search complexity of each particle and improves the overall performance of PSO. The equation of QPSO is:

(1) $$\left\{ \begin{array}{c} x_{id}^{t+1}=p_{id}^{t}\pm \beta \times \left|m{best}_{d}^{t}-x_{id}^{t}\right|\times \ln(1/u_{id}^{t})\\ p_{id}^{t}=\varphi_{id}^{t}\times P_{id}^{t}+(1-\varphi_{id}^{t})\times P_{gd}^{t}\\ m{best}_{d}^{t}=\{m{best}_{1}^{t},\cdots ,m{best}_{D}^{t}\}=\{\frac{1}{M}\sum_{i=1}^{M}P_{i1}^{t},\cdots ,\frac{1}{M}\sum_{i=1}^{M}P_{iD}^{t}\} \end{array}\right.$$

where $x_{id}^{t+1}$ is the position of particle *i* at (*t* + 1)-th iteration, i={1,⋯,M}, *M* is the size of particle swarm, *D* is the dimension of solution space, and $\varphi_{id}^{t}$ and $u_{id}^{t}$ are random numbers with uniform distribution between 0 and 1, respectively, If $\varphi_{id}^{t}\geq 0.5$, then take “+” for “±“ in formula $x_{id}^{t+1}$, otherwise take “−”. $p_{id}^{t}$ is the local gravitational factor, β=(1−0.5)×(T−t)/T+0.5 is called the contraction–expansion (CE) coefficient, $P_{id}^{t}$ is the local optimal solution of particle *i* in dimension *d* at *t*-th iteration, $P_{gd}^{t}$ is the global optimal solution in dimension *d* at *t*-th iteration, and $m{best}_{d}^{t}$ is the mean value of local optimal solutions for all particles in dimension *d* at *t*-th iteration.

The introduction of variable $m{best}_{d}^{t}$ improves the convergence rate and performance of the algorithm because it considers the distribution of the local optimal solution for all particles in the same dimension. In this paper, Equation (1) will be used as the solution equation for the adaptive generation and updating of radio fingerprint samples.

## 3. ULTF

### 3.1. The Principle of ULTF

RSS collecting is the key for off-line and online phase in fingerprint positioning. The mobile terminal is used to collect RSS, but the traditional offline acquisition process is inefficient. Nowadays, many mobile terminals are equipped with an acceleration sensor, a direction sensor, and gyroscopes. These sensors make it convenient to calculate the specific moving direction and acceleration of the user with a mobile terminal. Not only that, mobile terminals have powerful data interaction and processing capabilities. ULTF makes full use of this ability of the mobile terminal to collect RSS and other data in the positioning area autonomously. Then, all feedback data will be uploaded to the positioning system server. Finally, these data will be used for generating the user-location track. From the user entering the underground positioning area to leaving this area, an integrated user-location track will be recorded. Each user-location track contains many user-location track points. Each user-location track point contains the user relative position (this position cannot be directly used as the user’s estimated position) and received signal strength indication (RSSI) recorded at a certain time. QPSO algorithm will use the user relative position and RSSI to do the optimal radio fingerprint match.

### 3.2. User-Location TrackGeneration

**Figure 2:**
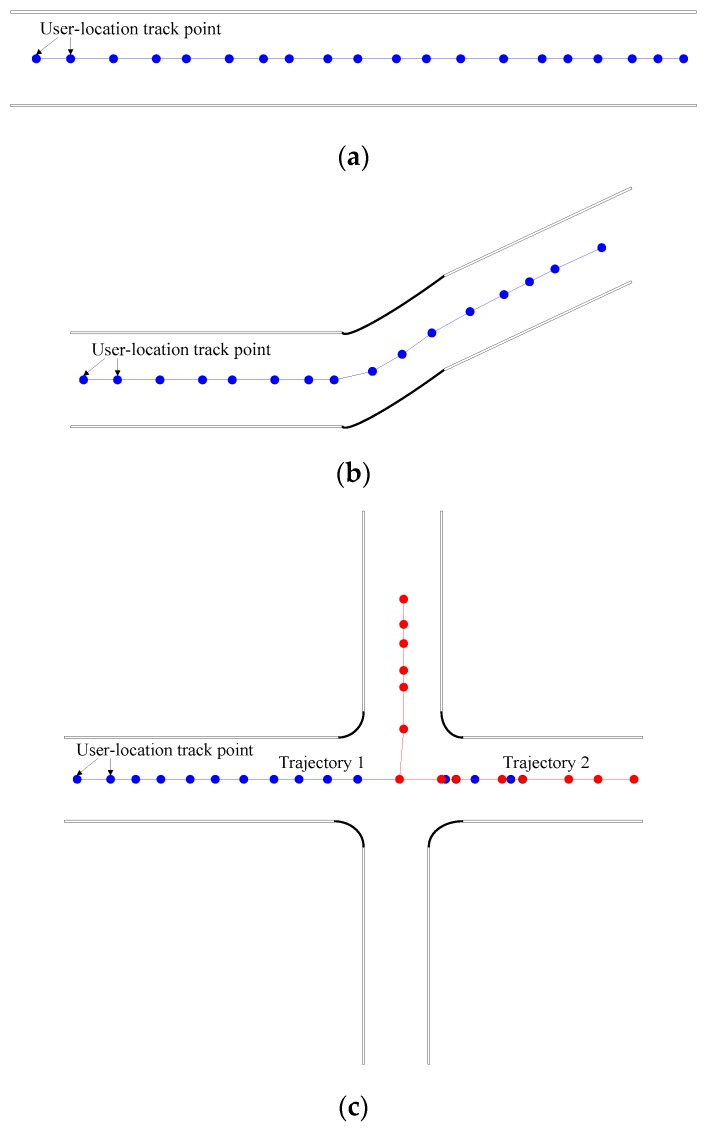
Diagram of user-location track: (**a**) straight-line tunnel; (**b**) irregular linear tunnel; (**c**) tunnel with crossing.

In general, the underground tunnel has a special spatial structure. The width and length of the tunnel are not the same order of magnitude. The length of the tunnel is several hundred meters, even several kilometers, but the width of the tunnel is only a few meters. Therefore, a miner’s movement in the tunnel width direction can practically be ignored. A sketch of a user-location track is shown in Figure 2. It is assumed that the user-location track points are always recorded at the middle position in the direction of tunnel width on the 2-D plane.

The user-location track generation is related to the user’s moving distance and direction. When the miners with mobile terminals walk in the tunnel, their movement direction and distance can be calculated through the data acquisition and processing of sensors in the mobile terminal. The different ways of holding a mobile phone may affect the calculation of the user’s moving distance. To solve this problem, this paper uses the step-by-step method to correct the user’s moving distance [15]. In this way, the user-location track can be accurately calculated.

## 4. Adaptive Radio Fingerprint Database

The construction and update of the adaptive radio fingerprint database are based on the combination of the ULTF and QPSO algorithms. ULTF is mainly responsible for the collection, upload, and calculation of the data related to the user-location track. These data include the user’s walking status and RSS at each sampling time. The user relative position calculated by ULTF in the user-location track point is used for the best reference point match. In the process of building and updating the adaptive radio fingerprint database, the QPSO algorithm is used for generating RSSI adaptively, but QPSO is used differently in the process of building and updating. Section 4.2 will illustrate the construction and update of the adaptive radio fingerprint database, respectively.

### 4.1. Adaptive Construction of RadioFingerprint Database Based on QPSO–ULTF

#### 4.1.1. The Principle and Process of QPSO–ULTF for Adaptive Construction of Radio Fingerprint Database

Each user-location track point generated by ULTF contains a feedback packet, and its data structure is $UF_{u}^{t}=({\mathrm{MAC}}_{u}:(TP_{u}^{t},f_{u}^{t}))$, where ${\mathrm{MAC}}_{u}$ is the WLAN MAC address of the mobile phone carried by user *u*, $TP_{u}^{t}$ is the data uploaded by user *u* to calculate the user’s location track point at time *t*, and $f_{u}^{t}$ is the real-time RSSI collected by user *u* at time *t.* With this feedback packet, the positioning system server will make the best match between user-location track points and reference points. The best match is mainly used to find the reference point with the most similar RSSI compared with the RSSI of the user-location track point. Then, the RSS generated by the QPSO algorithm will be stored in the radio fingerprint samples of this reference point. 

**Figure 3:**
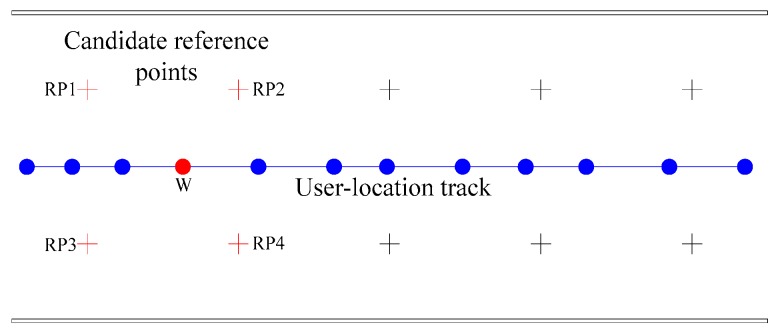
User-location track point and its corresponding candidate reference points.

To reduce the influence of accumulated error on the generation of radio fingerprint samples, it is necessary to select *PB* reference points closest to the user-location track point for the best reference point ${RP}_{best}$ matching. For example, the relative position between the user-location track and reference point is shown in Figure 3. If *PB* = 4, RP1, RP2, RP3, and RP4 will be the candidate reference points of ${RP}_{best}$ for user-location track point W. Because the RP1, RP2, RP3, and RP4 are the four nearest reference points to W, *PB* can also be regarded as the dimension of particle solution space in QPSO. The value of ${RP}_{best}$ is determined by the QPSO optimization process between the RSSI of user-location track points and the radio fingerprint calibration samples of reference points in each dimension.

In the off-line phase of most location fingerprints positioning systems, thousands of RSSI should be collected as the original radio fingerprint samples at each reference point. This phase is the most time-consuming and laborious part of the construction of an underground personnel positioning system. The method proposed in this paper also needs to manually collect the original samples as calibration samples, but the number of RSSI samples collected at each reference point can be reduced to 1/20 of the traditional original samples. The reason is that the QPSO–ULTF can generate a large set of samples from a small number of accurate radio fingerprint samples. The algorithm can improve the deployment efficiency of an underground personnel positioning system. In the QPSO algorithm, the variables to be solved are regarded as particles. The initially global optimal solution is generally the particle itself. The initially local optimal solution is also the particle itself in each dimension. For the application of QPSO in the adaptive construction of the radio fingerprint database, the $f_{u}^{t}$ in the feedback packet $UF_{u}^{t}$ of the user-location track point is regarded as the particle. The initially global optimal solution is $f_{u}^{t}$. The initially local optimal solution is $f_{u}^{t}$ in each dimension. In QPSO–ULTF, 200 sets of RSSI samples are collected at each reference point as calibration samples. The calibration samples are mainly used as the random correlation data for QPSO. During the process of QPSO iteration, the $f_{u}^{t}$ will be updated iteratively after being compared with the RSS mean value in the calibration samples. The number of RSSI involved in the QPSO operation in calibration samples is the total number of particles in the particle swarm.

The main steps of QPSO optimal radio fingerprint match and adaptive generation of RSSI are as follows.

Step 1. Initialize the QPSO parameter according to Equation (1). The number of particles is *M*. The dimension of solution space is *D*. The algorithm iteration is *T*. The constriction factor is β=(1.0−0.5)×(T−t)/T+0.5; *t* is the current iteration. The location track point is W. The real-time RSSI in the feedback data at W is $f_{W}=\{f_{{\mathrm{AP}}_{1}},\cdots ,f_{{\mathrm{AP}}_{k}},\cdots ,f_{{\mathrm{AP}}_{R}}\}$, where $f_{{\mathrm{AP}}_{k}}$ is the value of WiFi signal ${\mathrm{AP}}_{k}$, and *R* is the total number of WiFi signals. From all reference points, select *PB* (*PB*=*D*) reference points closest to W, whose location coordinates are ${RP}_{\rho }=(x_{\rho },y_{\rho })$, where ρ={1,⋯,PB}. Randomly select *M* consecutive RSSI samples $SF_{\rho }=\left\{sf_{\rho 1},\;\cdots ,sf_{\rho i},\cdots ,sf_{\rho M}\right\}$ from calibration samples of ρ−th candidate reference point as the correction sample sequences of *M* particles, where $sf_{\rho i}=\{sf_{\rho i}({\mathrm{AP}}_{1}),\cdots ,sf_{\rho i}({\mathrm{AP}}_{R})\}$. Then, the particle swarm on dimension ρ is $CF_{\rho }^{t}=\{cf_{\rho 1}^{t},\cdots ,cf_{\rho i}^{t},\cdots ,cf_{\rho M}^{t}\}$, where $cf_{\rho i}^{t}=\{f_{\rho i}^{t}({\mathrm{AP}}_{1}),$ $\cdots ,f_{\rho i}^{t}({\mathrm{AP}}_{k}),\cdots ,f_{\rho i}^{t}({\mathrm{AP}}_{R})\}$. The $cf_{\rho i}^{0}$ in initial value $CF_{\rho }^{0}$ of $CF_{\rho }^{t}$ are all $f_{w}$.

Step 2. At the *t*-th iteration, the local optimal solution $P_{\rho i}^{t}$ on dimension ρ is calculated by Equation (2). The global optimal solution $P_{g\rho }^{t}$ is calculated by Equation (3). Equation (4) is used to update $cf_{\rho i}^{t}$. This update does not affect the original calibration samples.

(2) $$\left\{ \begin{array}{c} P_{\rho i}^{t}=\left\{f_{\rho i}^{t}({\mathrm{AP}}_{k})|i=\arg\min{\left\|cf_{\rho i}^{t}-E(sf_{\rho i})\right\|}^{2},k=1,\cdots ,M\right\}\\ E(sf_{\rho i})=\{\frac{1}{M}\sum_{i=1}^{M}sf_{\rho i}({\mathrm{AP}}_{1}),\cdots ,\frac{1}{M}\sum_{i=1}^{M}sf_{\rho i}({\mathrm{AP}}_{R})\} \end{array}\right.$$

where ${\left\|\cdot \right\|}^{2}$ is the Euclidean distance between two vectors.

(3) $$P_{g\rho }^{t}=\{P_{\rho i}^{t}|\rho =\arg\min{\left\|P_{\rho i}^{t}-E(sf_{\rho i})\right\|}^{2}\}$$

(4) $$\left\{ \begin{array}{c} cf_{\rho i}^{t+1}=\{f_{\rho i}^{t+1}({\mathrm{AP}}_{1}),\cdots ,f_{\rho i}^{t+1}({\mathrm{AP}}_{k}),\cdots ,f_{\rho i}^{t+1}({\mathrm{AP}}_{R})\}\\ f_{\rho i}^{t+1}({\mathrm{AP}}_{k})=⟦p_{\rho i}^{t}({\mathrm{AP}}_{k})\pm \beta \times {\left\|f_{\rho i}^{t}({\mathrm{AP}}_{k})-E(sf_{\rho i}({\mathrm{AP}}_{k}))\right\|}^{2}\times \ln(1/u_{\rho i}^{t})⟧\\ p_{\rho i}^{t}({\mathrm{AP}}_{k})=\varphi_{\rho i}^{t}\times P_{\rho i}^{t}({\mathrm{AP}}_{k})+(1-\varphi_{\rho i}^{t})\times P_{g\rho }^{t} \end{array}\right.$$

where ⟦·⟧ stands for rounding in Equation (4), and the values of β, $\varphi_{\rho i}^{t}$, and $u_{\rho i}^{t}$ are the same as those in Equation (1).

Step 3. Set t=t+1. If t+1<T, return to *Step 2*. If t+1=T, terminate the iteration and output the global optimal solution $P_{g\rho }^{T}$ at iteration *T*. According to Equation (3), the candidate reference point corresponding to ρ in $P_{g\rho }^{T}$ is the best matching reference point.

After the above three main steps, the best matching reference point is found at a certain location track point, and a set of adaptive RSSI samples corresponding to the best matching reference point is generated. These generated RSSI samples of this best matching reference point will be stored in the radio fingerprint database. When there are enough user-location tracks, the RSSI samples generated by the QPSO–ULTF of each reference points reach a certain number. The RSSI samples can represent the RSS statistical distribution characteristics of each reference point. At that point, the construction of the underground WLAN adaptive radio fingerprint database is complete.

The positioning system will generate a large number of user-location track points during the process of the miners walking in the tunnel. There is a certain error between the positions of track points relative to *PB* candidate reference points and user-location track points. Meanwhile, there may be some interference in dynamic RSSI collected at the user-location track point. Therefore, it is necessary to determine whether the QPSO algorithm is applied to user-location track point W through Equation (5).

(5) $${\left\|f_{W}-E(sf_{\rho i})\right\|}^{2}\leq Q_{\mathrm{RSSI}},\;\rho =\{1,\cdots ,PB\}$$

where $E(sf_{\rho i})$ is the mean value of all $sf_{\rho i}$, and $Q_{\mathrm{RSSI}}$ is the RSS threshold.

**Figure 4:**
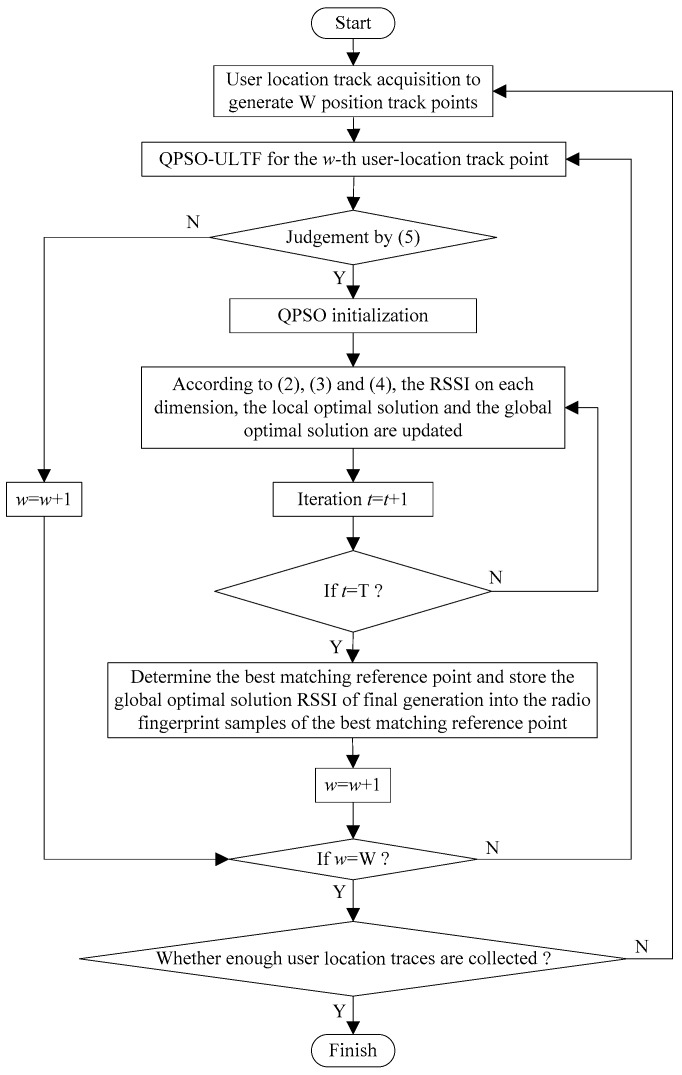
The flowchart of the adaptive construction method of radio fingerprint database based on QPSO–ULTF.

The flowchart of the adaptive construction method of radio fingerprint database based on QPSO–ULTF is shown in Figure 4.

#### 4.1.2. Experimental Comparison of the Adaptive Radio Fingerprint Database and Traditional Radio Fingerprint Database

To verify the reliability of the adaptive radio fingerprint database, a comparison of the statistical distribution characteristics of the samples in the manual radio fingerprint database and the adaptive radio fingerprint database is made, and then analyzed in the same underground positioning environment [16].

**Figure 5:**
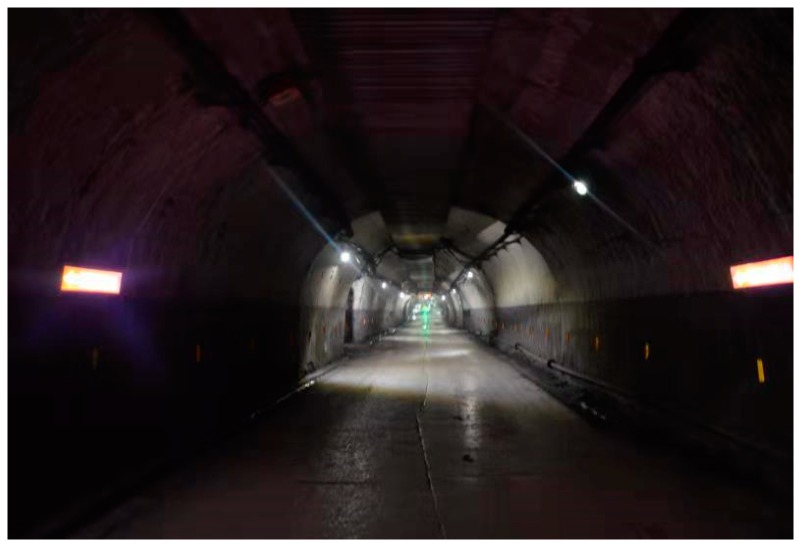
Underground tunnel for experiment.

**Figure 6:**
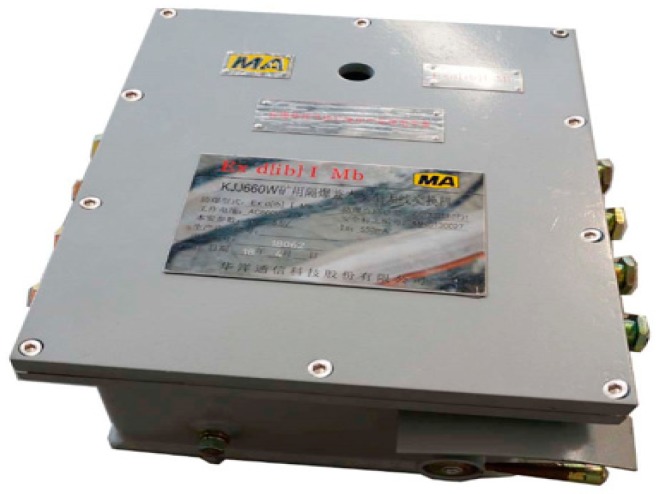
KJJ660W.

**Figure 7:**
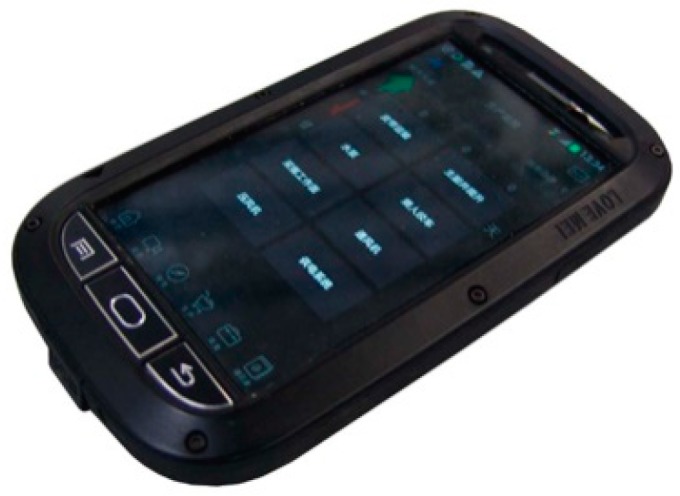
The intrinsic safety mobile phone.

**Figure 8:**
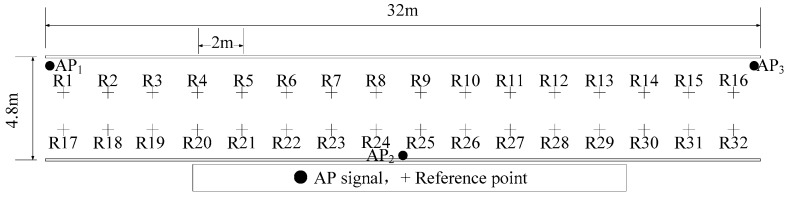
Plan of experiment tunnel with radio fingerprint database comparison.

The underground tunnel for the experiment is shown in Figure 5. The access point (AP) device used in all experiments of this paper is KJJ660W, which is shown in Figure 6. The mobile device is shown in Figure 7, its operating system is Android 6.0. As shown in Figure 8, there are three AP signals and 32 reference points in the positioning tunnel. The manual radio fingerprint database collects 3000 RSSI at each reference point, while the adaptive radio fingerprint database only collects 200 RSSI. In the process of building the adaptive radio fingerprint database, each one of the three miners carries a different intrinsic safety mobile phone and walks in the positioning tunnel 1000 times. This forms 3000 user-location tracks. The adaptive radio fingerprint database is constructed from 3000 user-location tracks and QPSO algorithm. Initialize the parameters, *T* = 20, *PB* = 4, *M* = 200, $Q_{\mathrm{RSSI}}=7.348$, ${\mathrm{QUA}}_{\mathrm{LT}}=2000$, and $Q_{\mathrm{LTP}}^{\mathrm{EX}}=0.75$.

**Figure 9:**
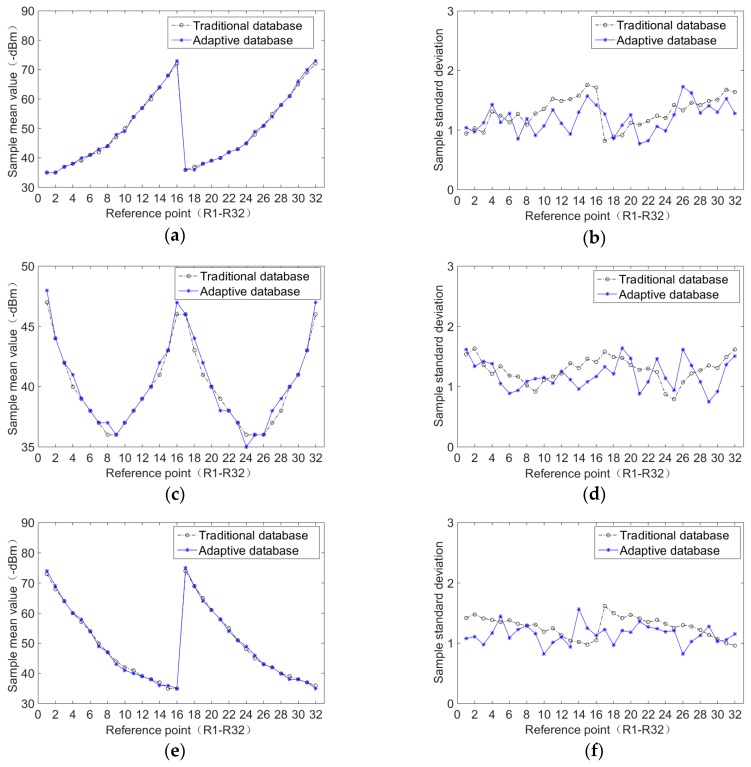
Comparison of radio fingerprint samples statistical distribution between the adaptive radio fingerprint database and the traditional radio fingerprint database: (**a**) mean value of ${\mathrm{AP}}_{1}$; (**b**) standard deviation of ${\mathrm{AP}}_{1}$; (**c**) mean value of ${\mathrm{AP}}_{2}$; (**d**) standard deviation of ${\mathrm{AP}}_{2}$; (**e**) mean value of ${\mathrm{AP}}_{3}$; (**f**) standard deviation of ${\mathrm{AP}}_{3}$.

Figure 9 shows the comparison of the statistical characteristic distribution between the adaptive radio fingerprint database and the traditional radio fingerprint database. Compared with the samples in the traditional radio fingerprint database, the mean value of RSSI received from three AP signals is similar, and the standard deviation of RSSI is also in a small range. In other words, the adaptive radio fingerprint database can replace the traditional radio fingerprint database. In addition, due to the reduction in the demand for the number of samples collected manually, the resource consumption of manpower and time is also reduced when building the adaptive radio fingerprint database. This has important theoretical significance and application value for the comprehensive research of underground WLAN personnel positioning systems.

### 4.2. Adaptive Update of RadioFingerprint Database Based on QPSO–ULTF

Some tunnel environmental noise will affect the statistical distribution of RSS at reference points. Due to this, the accuracy of some original samples in the radio fingerprint database may be decreased. Therefore, these samples need to be updated. When the location area is too large with many reference points, however, it is difficult to determine which reference points need to be updated. In many cases, it can only update and collect the RSSI for most or even all reference points. Not only is it time-consuming and laborious, but it also seriously affects the normal operation of the underground personnel positioning system.

In the adaptive construction of the radio fingerprint database in Section 4.1, Equation (5) determines the real-time RSSI of each user-location track point to ensure that the RSSI calculated by QPSO is similar samples without large noise interference compared with the calibration samples of candidate reference points. However, if the real-time RSSI of continuous user-location track points in multiple user-location tracks do not satisfy Equation (5) in a period of time, it is possible that the change of location area environment has caused the change in RSS statistical characteristic distribution. It makes the real-time RSSI collected near the candidate reference points differ substantially from the calibration samples of the candidate reference points. Therefore, by analyzing and processing the abnormal data in multiple user-location trajectory feedback information, we can find the positions of the reference points where the RSS distribution changed. At that point, the calibration samples at these reference points should be updated.

#### 4.2.1. RSS Distribution When Tunnel Environment Changes

**Figure 10:**
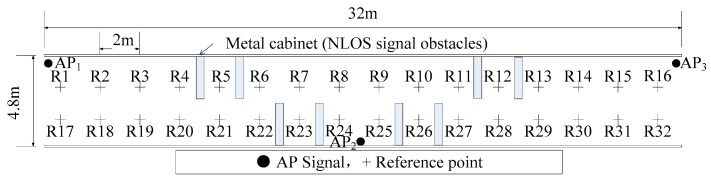
Plan of tunnel with non-line of sight (NLOS) signal obstacles.

In order to analyze the change in RSS distribution between $f_{u}^{t}$ in $UF_{u}^{t}$ and the calibration samples of the candidate reference points, eight metal cabinets, each with a length of 2.2 m, a width of 0.5 m, and a height of 2.1 m are placed in the tunnel, as shown in Figure 10.

**Figure 11:**
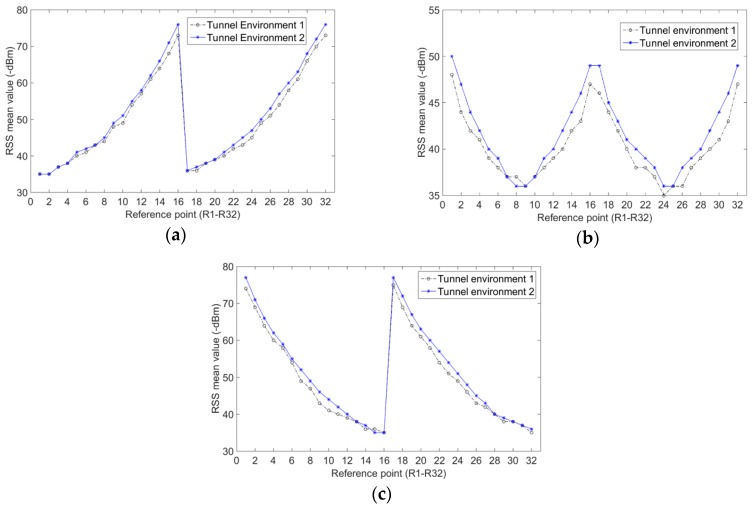
Received signal strength (RSS) mean value comparison of each reference point when tunnel environments changed: (**a**) mean value of ${\mathrm{AP}}_{1}$; (**b**) mean value of ${\mathrm{AP}}_{2}$; (**c**) mean value of ${\mathrm{AP}}_{3}$.

Figure 11 shows the comparison of RSS mean value at each reference point after the tunnel environment has changed. Of these, Environment 1 is shown in Figure 8, and Environment 2 is shown in Figure 10. The results of Environment 1 are the RSS mean values of the radio fingerprint samples calculated in the experiments in Section 4.1.2. The results of Environment 2 are the RSS mean values collected from 1000 sets of RSSI at each reference point. The results of the comparison, as seen in Figure 11, show that the change of tunnel environments brings about the change of RSS mean value at some reference points. If the radio fingerprint database that is adaptively constructed in Environment 1 is still used to locate the miners, it is bound to make a large positioning error.

#### 4.2.2. The Principle and Process of QPSO–ULTF for Adaptive Update of Radio Fingerprint Database

**Figure 12:**
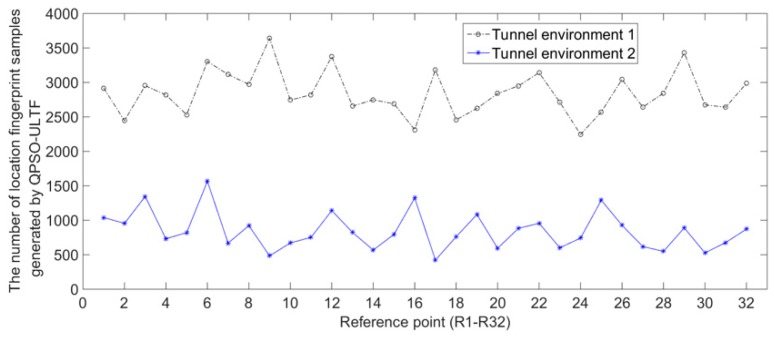
Quantity of radio fingerprint samples generated by the QPSO–ULTF in different tunnel environments.

**Figure 13:**
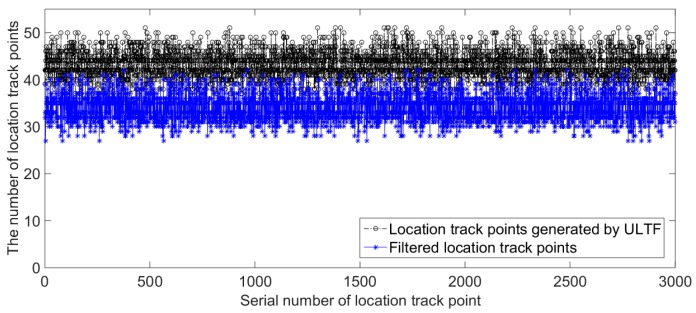
Quantitative comparison of filtered location track points after tunnel environment changed.

According to the analysis in Section 4.2.1, the RSS distribution at most reference points will change when the location environments change. There will be a big difference between the $f_{u}^{t}$ in feedback data of user-location track points and the RSS mean values of calibration samples of candidate reference points. Most of the feedback data of user-location track points are not satisfied with Equation (5). We can observe that the number of user-location track points have filtered out what they are not satisfied with Equation (5) in experiments. Figure 12 gives the number of radio fingerprint samples generated by the QPSO–ULTF at each reference point in the two tunnel environments. The experimental data collection process is similar to the process explained in Section 4.1.2. The adaptive radio fingerprint database is constructed by 3000 user-location tracks of three miners. The calibration samples of each reference point are collected in tunnel Environment 1. The comparison between the number of user-location track points and the number of user-location track points that do not satisfied with Equation (5) is shown in Figure 13. In Environment 1,126,742 location track points are recorded, and 91,031 radio fingerprint samples are generated adaptively from 32 reference points. In Environment 2, 122,945 location track points are recorded, and 27,036 radio fingerprint samples are generated adaptively from 32 reference points. The comparison results in Figure 13 show that 77% of the location track points are filtered out because they do not meet the criteria of the QPSO–ULTF operation after the tunnel environment changed. Therefore, considering that the change in RSS distribution at reference point caused by the change of tunnel environment is generally happened. A scale threshold can be set as a criterion to determine whether the location environment has changed.

In general, once the adaptive radio fingerprint database is constructed, the QPSO–ULTF operation will not be performed on user-location track points. Due to the necessity to monitor the RSS distribution in a tunnel environment, the comparison process between $f_{u}^{t}$ in the feedback data of user-location track points and the statistical mean $E(sf_{\rho i})$ of calibration samples in candidate reference points still need to be carried out continuously. In addition, the data of all filtered user-location track points are stored during the comparison process. To improve the examined ability and accuracy of the personnel positioning system once the tunnel environment has changed, Equation (5) needs to be modified.

Because the miners go in and out of the mine in teams, there will be different users going in and out of the positioning area at different time intervals. In addition, the number of miners in each team is not the same. So, in order to better record and differentiate the user-location tracks, it is necessary to add time stamp ${\mathrm{TIME}}_{u}$ into the user-location trajectory feedback packet. Accordingly, $UF_{u}^{t}$ is modified to $UF_{u}^{t}=({\mathrm{MAC}}_{u}:(TP_{u}^{t},f_{u}^{t}):{\mathrm{TIME}}_{u})$. The introduction of time stamp is useful for analyzing the proportion of location track points that are filtered better at a specific time interval.

According to Equation (5), the filtering condition of location track points is:

(6) $${\left\|f_{W}-E(sf_{\rho i})\right\|}^{2}>Q_{\mathrm{RSSI}},\;\rho =\{1,\cdots ,{PB}_{\mathrm{EX}}\}$$

where the definitions of $f_{W}$, $E(sf_{\rho i})$, and $Q_{\mathrm{RSSI}}$ are the same as those in Equation (5). ${PB}_{\mathrm{EX}}$ is the number of reference points closest to the user-location track point for data comparison when determining whether the user-location track point is filtered or not; its value usually sets to 2. 

If $f_{W}$ of location track point is satisfied with Equation (6) for the nearest ${PB}_{\mathrm{EX}}$ reference points, the relevant data of this track point will be recorded as $W_{\mathrm{EX}}=\{{\mathrm{MAC}}_{u},f_{W},{EX}_{\mathrm{RP}},{\mathrm{TIME}}_{u}\}$, where ${EX}_{\mathrm{RP}}=\{{\mathrm{RP}}_{Num1},\cdots ,{\mathrm{RP}}_{Num{PB}_{\mathrm{EX}}}\}$ is the serial number of ${PB}_{\mathrm{EX}}$ reference points closest to this filtered track point.

Therefore, the basis for determining the change of positioning area environments is as follows:

(7) $$\left\{ \begin{array}{c} {Qua}_{\mathrm{LT}}\geq {\mathrm{QUA}}_{\mathrm{LT}}\\ \mathrm{and}\\ {Qua}_{\mathrm{LTP}}^{\mathrm{EX}}/{Qua}_{\mathrm{LTP}}\geq Q_{\mathrm{LTP}}^{\mathrm{EX}} \end{array}\right.$$

where ${Qua}_{\mathrm{LT}}$ is the number of user-location tracks generated in a specific period ${\mathrm{Time}}_{\mathrm{EX}}$, ${\mathrm{QUA}}_{\mathrm{LT}}$ is the given number of user-location tracks, ${Qua}_{\mathrm{LTP}}^{\mathrm{EX}}$ and ${Qua}_{\mathrm{LTP}}$ are, respectively, the number of filtering and generation of user-location track points in a specific period ${\mathrm{Time}}_{\mathrm{EX}}$, and $Q_{\mathrm{LTP}}^{\mathrm{EX}}$ is the percentage of filtered location track points. ${Qua}_{\mathrm{LT}}$, ${Qua}_{\mathrm{LTP}}^{\mathrm{EX}}$, and ${Qua}_{\mathrm{LTP}}$ will change dynamically over time. The record of their value is determined by the current system time, ${\mathrm{Time}}_{\mathrm{EX}}$ and ${\mathrm{TIME}}_{u}$.

When Equation (7) is satisfied, the environment of positioning area changes and the radio fingerprint database needs to be updated dynamically. Because the existing radio fingerprint samples can no longer be used for precise positioning, most of the radio fingerprint samples of reference points need to be rebuilt. However, the calibration samples of reference points cannot be used for QPSO calculation in the adaptive generation of radio fingerprint samples. As shown in Figure 1, calibration samples should be updated first. Overall, QPSO for the adaptive update of the radio fingerprint database mainly updates the calibration samples of each reference point. The RSSI in the filtered user-location track points is used as the reference for updating calibration samples. Similar to the best match between user-location track points and reference points in the adaptive construction of the radio fingerprint database, each filtered user-location track point also has its corresponding filtering candidate reference points.

The update steps are as follows.

Step 1. First, the filtered user-location track points are clustered. If the ${EX}_{\mathrm{RP}}$ in the record data $W_{\mathrm{EX}}$ of filtered user-location track points are the same, the filtered user-location track points with the same ${EX}_{\mathrm{RP}}$ shall be classified as $W_{\mathrm{EX}}({EX}_{\mathrm{RP}})$.

Step 2. To reduce the noise interference in RSSI, LQI is used to filter the RSSI with noise in user-location track points after clustering [17]. The filtered $W_{\mathrm{EX}}({EX}_{\mathrm{RP}})$ is recorded as ${\tilde{W}}_{\mathrm{EX}}({EX}_{\mathrm{RP}})$.

Step 3. The RSSI in ${\tilde{W}}_{\mathrm{EX}}({EX}_{\mathrm{RP}})$ mainly correspond to ${PB}_{\mathrm{EX}}$ filtering candidate reference points ${EX}_{\mathrm{RP}}$. Suppose that ${\tilde{W}}_{\mathrm{EX}}({EX}_{\mathrm{RP}})$ contains $M_{\mathrm{EX}}$ filtered user-location track points, then their corresponding RSSI are $\left\{f_{W}^{1},\cdots ,f_{W}^{r},\cdots ,f_{W}^{M_{\mathrm{EX}}}\right\}$ and $f_{W}^{r}=\{f_{W}^{r}({\mathrm{AP}}_{1}),\cdots ,f_{W}^{r}({\mathrm{AP}}_{k}),\cdots ,f_{W}^{r}({\mathrm{AP}}_{R})\}$. Filtered candidate reference points are ${EX}_{\mathrm{RP}}=\{{\mathrm{RP}}_{Num1},\cdots ,{\mathrm{RP}}_{Numl},\cdots ,{\mathrm{RP}}_{Num{PB}_{\mathrm{EX}}}\}$. The iteration number of algorithm is *T*, and a QPSO operation is performed on each filtered user-location track point in turn. The number of particles in particle swarm is *M*. The solution space dimension is $D={PB}_{\mathrm{EX}}$. $\rho =\{1,\cdots ,{PB}_{\mathrm{EX}}\}$. The original calibration samples of filtered candidate reference point ${\mathrm{RP}}_{Numl}$ is $SF_{\rho }=\left\{sf_{\rho 1},\;\cdots ,sf_{\rho i},\cdots ,sf_{\rho M}\right\}$, where $sf_{\rho i}=\{sf_{\rho i}({\mathrm{AP}}_{1}),\cdots ,sf_{\rho i}({\mathrm{AP}}_{k}),\cdots ,sf_{\rho i}({\mathrm{AP}}_{R})\}$.

Step 4. For the *r*-th filtered user-location track point at the *t*-th iteration, the local optimal solution $P_{\rho i}^{t}$ on dimension ρ is calculated by Equation (8), and the global optimal solution $P_{g\rho }^{t}$ is calculated by Equation (9). Equation (10) is used to update $sf_{\rho i}^{t}$ for getting $sf_{\rho i}^{t+1}$ at (*t* + 1)-th iteration.

(8) $$\left\{ \begin{array}{c} P_{\rho i}^{t}=\left\{sf_{\rho i}^{t}({\mathrm{AP}}_{k})|i=\arg\min{\left\|sf_{\rho i}^{t}-E(f_{W}^{r})\right\|}^{2},k=1,\cdots ,M\right\}\\ E(f_{W}^{r})=\{\frac{1}{M_{\mathrm{EX}}}\sum_{i=1}^{M_{\mathrm{EX}}}f_{W}^{r}({\mathrm{AP}}_{1}),\cdots ,\frac{1}{M_{\mathrm{EX}}}\sum_{i=1}^{M_{\mathrm{EX}}}f_{W}^{r}({\mathrm{AP}}_{R})\} \end{array}\right.$$

(9) $$P_{g\rho }^{t}=\{P_{\rho i}^{t}|\rho =\arg\min{\left\|P_{\rho i}^{t}-E(f_{W}^{r})\right\|}^{2}\}$$

(10) $$\left\{ \begin{array}{c} sf_{\rho i}^{t+1}=\{sf_{\rho i}^{t+1}({\mathrm{AP}}_{1}),\cdots ,sf_{\rho i}^{t+1}({\mathrm{AP}}_{k}),\cdots ,sf_{\rho i}^{t+1}({\mathrm{AP}}_{R})\}\\ sf_{\rho i}^{t+1}({\mathrm{AP}}_{k})=⟦p_{\rho i}^{t}({\mathrm{AP}}_{k})\pm \beta \times {\left\|sf_{\rho i}^{t}({\mathrm{AP}}_{k})-E(f_{W}^{r}({\mathrm{AP}}_{k}))\right\|}^{2}\times \ln(1/u_{\rho i}^{t})⟧\\ p_{\rho i}^{t}({\mathrm{AP}}_{k})=\varphi_{\rho i}^{t}\times P_{\rho i}^{t}({\mathrm{AP}}_{k})+(1-\varphi_{\rho i}^{t})\times P_{g\rho }^{t} \end{array}\right.$$

Step 5. Set t=t+1. If t+1<T, return to *Step 4*. If t+1=T, the reference point number *Numl* of the filtered candidate reference point ${\mathrm{RP}}_{Numl}$ corresponding to ρ is the reference point for updating the calibration samples. At this time, the global optimal solution $P_{g\rho }^{T}$ is used as the updated calibration samples of this reference point. It will be stored in the new calibration sample set $SF_{Numl}^{\mathrm{NEW}}$. Then, proceed to *Step 6*.

Step 6. Set r=r+1. If $r+1\leq M_{\mathrm{EX}}$, set *t* = 1, and return to *Step 4*. If $r+1>M_{\mathrm{EX}}$, then an adaptive radio fingerprint update is completed for filtered user-location track points $W_{\mathrm{EX}}({EX}_{\mathrm{RP}})$, corresponding to the reference point ${EX}_{\mathrm{RP}}$.

Step 7. Repeat *Steps 2* to *6* for all classified filtered location track points $W_{\mathrm{EX}}({EX}_{\mathrm{RP}})$. Finally, the original calibration samples *SF* is replaced by the newly generated calibration samples ${SF}^{\mathrm{NEW}}$ at each reference point. If the number of generated calibration samples at some reference points is small or none, the original calibration samples remain. The adaptive update process of the radio fingerprint database is finished.

The separation of training sets and test sets in radio maps are determined by the main parameters set by QPSO–ULTF, including *PB*, ${PB}_{\mathrm{EX}}$ and the number of calibration samples. After the adaptive update of the radio fingerprint database, it is also necessary to again perform the adaptive construction of the radio fingerprint database. The construction process is the same as the method proposed in Section 4.1.1.

**Figure 14:**
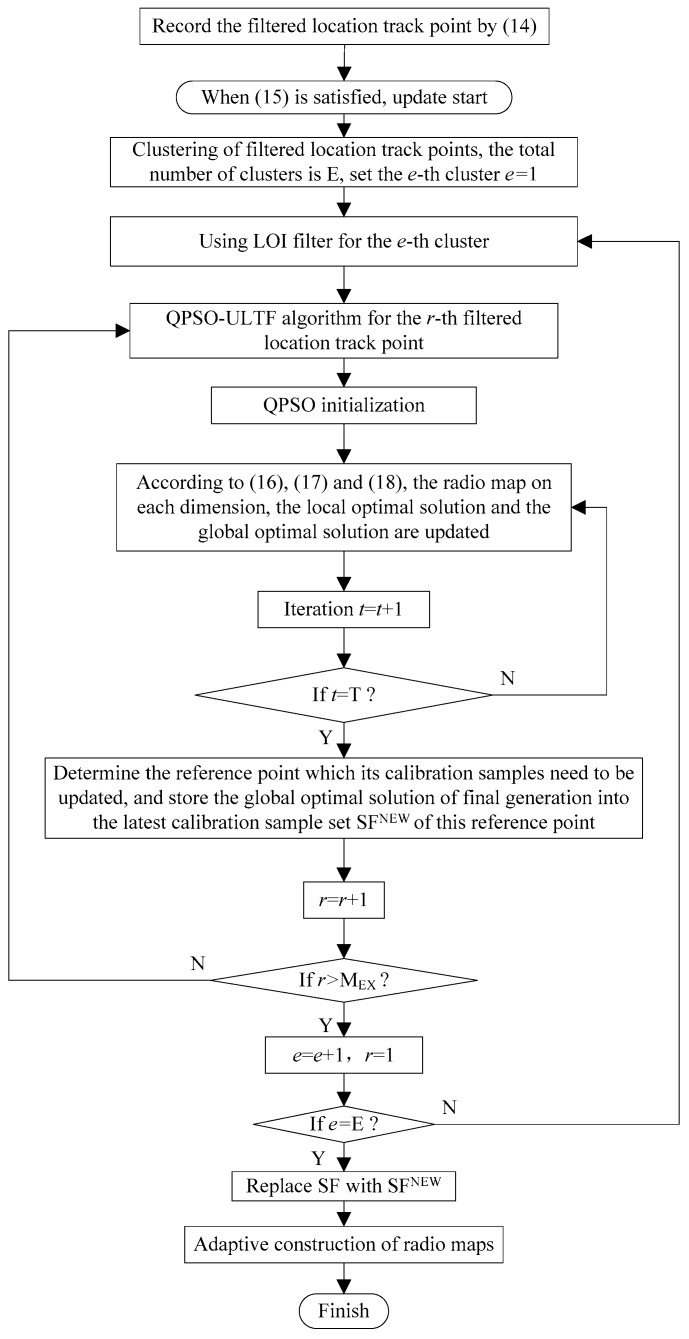
The flowchart of the adaptive update method of the radio fingerprint database based on QPSO–ULTF.

The flowchart of the adaptive update method of radio fingerprint database based on QPSO–ULTF is shown in Figure 14.

#### 4.2.3. Adaptive Updating Experiment of Radio Fingerprint Database

The tunnel plan for the adaptive updating experiment of the radio fingerprint database is shown in Figure 10. The original calibration samples are collected from the tunnel environment shown in Figure 8. The miner walks randomly 1000 times in the tunnel of Figure 10. Initialize the parameters, *T* = 20, *PB* = 4, ${PB}_{\mathrm{EX}}=2$, *M* = 200, $Q_{\mathrm{RSSI}}=7.348$, ${\mathrm{QUA}}_{\mathrm{LT}}=1000$, and $Q_{\mathrm{LTP}}^{\mathrm{EX}}=0.7$.

**Figure 15:**
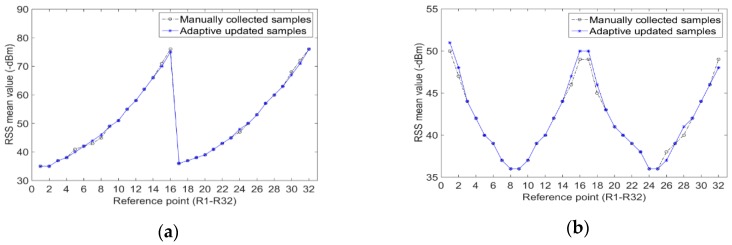

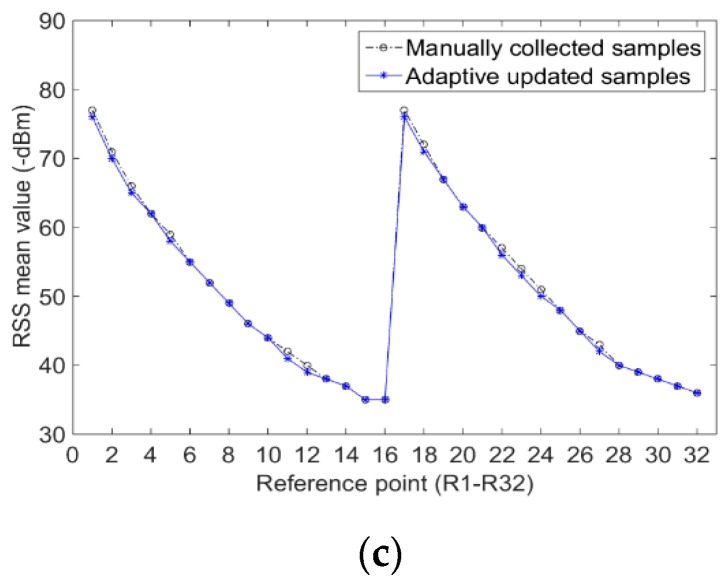
Of adaptive updated results of the radio fingerprint samples: (**a**) mean value of ${\mathrm{AP}}_{1}$; (**b**) mean value of ${\mathrm{AP}}_{2}$; (**c**) mean value of ${\mathrm{AP}}_{3}$.

Figure 15 shows the RSS mean value comparison results between 1000 groups of RSSI collected manually and RSSI generated by adaptive update at each reference point in the tunnel environment, as shown in Figure 10. After updating, the mean value of RSS calibration samples at each reference point is basically the same as the mean value of manually collected RSSI. It can be inferred that the adaptive updating of the radio fingerprint database based on QPSO–ULTF can achieve the better adaptive correction of RSS distribution at each reference point. The specific positioning experiments will be discussed in Section 4.

The value of $Q_{\mathrm{RSSI}}$ in Equation (6) is the main parameter that affects the updating performance of the radio fingerprint database. To analyze the influence of $Q_{\mathrm{RSSI}}$ on the adaptive updating process of radio fingerprints, the number of filtered user-location track points is recorded and compared with different values of $Q_{\mathrm{RSSI}}$. The initial data of experiments show 122,945 user-location track points of information generated by the miner who walked 3000 times in the tunnel environment, as shown in Figure 10. Four values of $Q_{\mathrm{RSSI}}$ are used for adaptive updating of the radio fingerprint database. The four values of $Q_{\mathrm{RSSI}}$ are $\sqrt{2\times R\times 1^{2}}$, $\sqrt{2\times R\times 2^{2}}$, $\sqrt{2\times R\times 3^{2}}$, and $\sqrt{2\times R\times 4^{2}}$. The number of AP signals *R* is three. The other parameter ${PB}_{\mathrm{EX}}$ is two.

**Figure 16:**
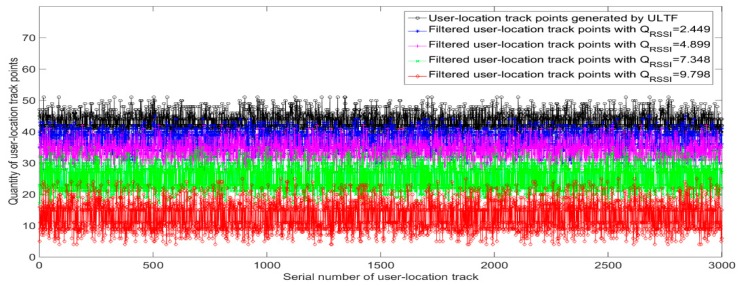
Quantitative comparison of filtered location track points with different $Q_{\mathrm{RSSI}}$.

Figure 16 shows the comparison of the quantity of filtered user-location track points in all user-location tracks with different values of $Q_{\mathrm{RSSI}}$. The total number of filtered user-location track points is 11,1491, 95,050, 75,231, and 39,940 when $Q_{\mathrm{RSSI}}$ is 2.449,4.899, 7.348, and 9.798, respectively. When $Q_{\mathrm{RSSI}}$ is large, only 32% of user-location track points are filtered out even if the positioning environment has changed. This will lead to a situation in which the system cannot accurately determine whether the tunnel environment is indeed changed. When $Q_{\mathrm{RSSI}}$ is small, nearly all the user-location track points are judged to be filtered out, which causes an increase in the load of the positioning system. In practical applications, it is necessary to choose a reasonable value. This should not only ensure that the observing sensitivity of underground positioning environment has changed, but should also consider the working efficiency of the positioning system. At the same time, it also should take into account the number of user-location track points filtered by the QPSO–ULTF when the radio fingerprint database is updated adaptively.

## 5. Results and Discussion

### 5.1. Establishment of Underground Positioning Experiment

This section mainly verifies the positioning performance of the adaptive radio fingerprint database in the tunnel. The experiments are divided into two groups: Experiment 1 and Experiment 2. The tunnel environments of the two groups are different. The AP device used in those two experiments is KJ660W which shown in Figure 6. The mobile device is an intrinsic safety mobile phone which shown in Figure 7.

Experiment 1 is aimed at verifying the positioning performance of an adaptive radio fingerprint database. The environment of this experimental tunnel is shown in Figure 5 and Figure 8. There are three AP signals and 32 reference points in the positioning tunnel. The two databases used in this experiment are the traditional radio fingerprint database constructed manually in Section 4.1.2 and the database constructed adaptively. The miners carry out 500 positioning tests for each of these two databases. In order to achieve comparable results, Weight K-Nearest Neighbors (WKNN) [18], Local Discriminant Embedding (LDE) [19,20], and Kernel Principal Component Analysis (KPCA) [21,22] are used to doing the positioning test.

Experiment 2 is aimed at verifying the positioning performance of the radio fingerprint database updated adaptively. The environment of this experimental tunnel is shown in Figure 10. As in Experiment 1, the two databases used in Experiment 2 are the traditional radio fingerprint databases constructed manually and the database updated adaptively which environment changed from Figure 8, Figure 9 and Figure 10. The underground personnel positioning system records and processes the positioning data of miners 500 times as they walked. Similar to Experiment 1, WKNN, LDE, and KPCA are also used for positioning tests.

### 5.2. Experimental Results and Analysis

**Figure 17:**
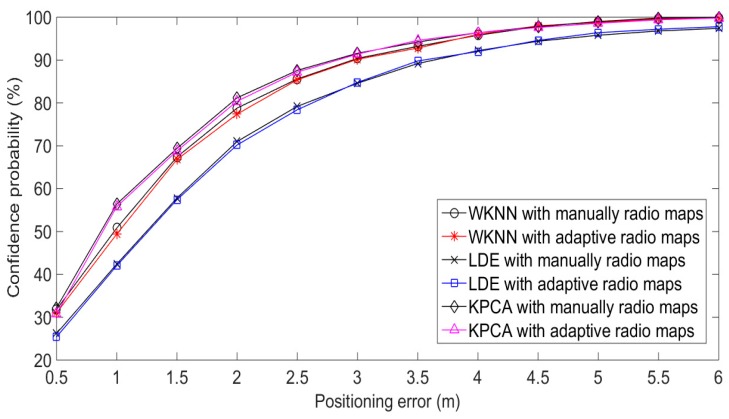
Dynamic positioning results of Experiment 1.

The positioning results of Experiment 1 are shown in Figure 17 and Table 1. The results in Figure 17 show that the adaptive radio fingerprint database has similar positioning performance with the manually constructed database, and the positioning errors are similar when the confidence probability is greater than 80%. For three positioning algorithms in Table 1, the positioning errors with confidence probability 90% of two radio fingerprint databases are basically the same. The construction of the adaptive radio fingerprint database only needs a small number of calibration samples, which effectively reduces the complexity of the process of off-line location fingerprint acquisition.

**Figure 18:**
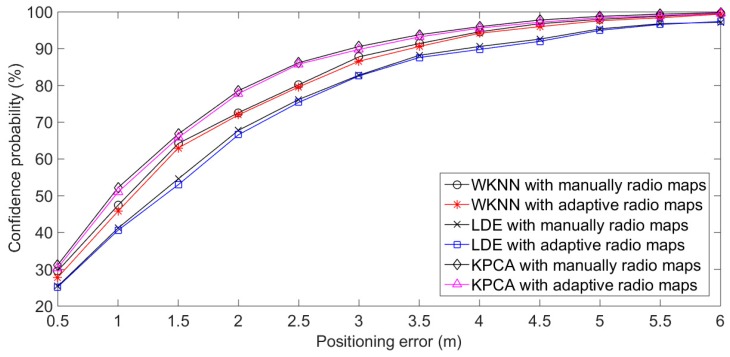
Dynamic positioning results of Experiment 2.

The results in Figure 18 and Table 2 show that the adaptive update of radio fingerprints is better able to capture the change in RSS distribution in the positioning environment, and that the positioning performance of radio fingerprint database updated adaptively can also be guaranteed. Although the positioning errors of LDE and KPCA for adaptive radio maps are 0.5 m larger than that of manually radio maps, these errors are acceptable in the application of underground personnel positioning. Moreover, the process of adaptive updating is completely processed by the underground personnel positioning system. Although this update process takes quite a long time for the positioning system (in Experiment 2, it took 1 h, 23 min, and 36 s), the radio fingerprint database can be reasonably updated in the relatively low-frequency period of time when the miners enter or exit the underground positioning area. The superior performance of the adaptive radio fingerprint database is due to the efficient search ability of the potential optimal solution in a multi-dimensional solution space by QPSO, as well as the dynamic extraction ability of particle swarm for individual and overall features of large samples of data.

## 6. Conclusions

In view of the tedious construction process of a WLAN radio fingerprint database, an adaptive construction and update method of radio fingerprint database based on the QPSO–ULTF is proposed.

The miners’ walking trajectories are recorded in real-time by the miners’ personal positioning mobile terminal. The data $UF_{u}^{t}$ collected at each time are uploaded to the personnel positioning system server. The system calculates and generates the user-location track according to these feedback packets, and all user-location track points in user-location tracks have their own reference points, which are relatively close to each other. According to the ULTF algorithm, the user-location track points that may contain noise are filtered out, and the filtered user-location track points may be an important parameter for adaptive updating of the radio fingerprint database. Then, by using the quantum properties in the process of particle dynamic search in QPSO, through the multi-dimensional feature learning with small samples of radio fingerprint, the potential global optimal solution is quickly found in the solution space, so as to complete the adaptive generation of radio fingerprints. 

Due to the peculiarity of coal mine production scheduling, there will be a certain number of miners entering or exiting the positioning area in a specific time period. Hence, the user-location trajectory can be recorded by a miner’s mobile terminal when the miner is walking in the tunnel. If the number of miners is large, in a short period of time, the user-location tracks will be recorded to achieve the requirements for building the radio fingerprint database. To reduce the impact of the update process on the normal operation of the underground personnel positioning system, the adaptive update of the radio fingerprint database can be carried out in the period of relatively low frequency when miners are entering or exiting the underground positioning area. 

It can be inferred from the experiment results that the adaptive radio fingerprint database constructed by the QPSO–ULTF has similar positioning performance with the traditional manual database, and it is better able to adaptively adjust to changes in the tunnel environment.

## Figures and Tables

**Table 1:** Positioning error with confidence probability 90% of three algorithms for two different databases in Experiment 1.

Database	WKNN	LDE	KPCA
Manually radio maps	3 m	4 m	3 m
Adaptive radio maps	3 m	4 m	3 m

**Table 2:** Positioning error with confidence probability 90% of three algorithms for two different databases in Experiment 2.

Database	WKNN	LDE	KPCA
Manually radio maps	3.5 m	4 m	3 m
Adaptive radio maps	3.5 m	4.5 m	3.5 m
